# Predictors of abnormal electroencephalogram and neuroimaging in children presenting to the emergency department with new-onset afebrile seizures

**DOI:** 10.1186/s12887-022-03668-6

**Published:** 2022-10-27

**Authors:** Noman Ali, Sohaib Haider, Syed Mustahsan, Murk Shaikh, Ahmed Raheem, Salman Muhammad Soomar, Shahan Waheed

**Affiliations:** 1 Department of Emergency Medicine, Aga Khan University, 74800 Karachi, Pakistan; 2 Department of Pediatrics & Child Health, Aga Khan University, 74800 Karachi, Pakistan

**Keywords:** Predictors, Neuroimaging, Pediatrics, Emergency, Afebrile seizures

## Abstract

**Background:**

Electroencephalogram and neuroimaging in pediatric patients with new-onset afebrile seizures are performed to detect any underlying pathological severe condition that may require emergent neuro-intervention and guide prognosis. This study aims to determine the predictors of abnormal EEG and neuroimaging in children presenting to the emergency department with new-onset afebrile seizures.

**Methods:**

This single-center cross-sectional study was conducted at a tertiary care hospital in Karachi, Pakistan, from July 01, 2019, to June 30, 2021. All patients aged one month to 18 years who presented with new-onset afebrile seizures were included. Demographic and clinical data were recorded, including age, gender, seizure type, duration of seizure, associated signs and symptoms, and disposition. Multivariable regression analysis was applied to determine the predictors of abnormal EEG and CT scan or MRI findings.

**Results:**

Out of 201 participants, most patients were in the infantile age group (41.3%), with an equal gender distribution. The most common type of seizure was generalized onset 152 (75.6%). EEG was performed on a total of 126 patients (62.7%) and out of these patients, 67 patients (53.1%) had abnormal findings. In a multivariable analysis, the age group of 5 to 10 years and seizure duration of more than 5 min were significantly associated with higher odds of abnormal EEG findings. In contrast, only the focal onset of seizure was significantly associated with higher odds of abnormal neuroimaging findings.

**Conclusion:**

The study emphasizes the need for a protocol regarding the performance of EEG and neuroimaging in children presenting to the ED with new-onset afebrile seizures that would aid emergency physicians in the direction of appropriate management, thus ensuring a better quality of patient care and outcomes.

**Supplementary Information:**

The online version contains supplementary material available at 10.1186/s12887-022-03668-6.

## Introduction

A seizure is a brief change in regular electrical activity in the brain resulting in alterations in awareness, perception, behavior, or movement [1]. It occurs in approximately 4 − 10% of children and accounts for 1% of all emergency department (ED) visits [2]. Seizures are broadly classified into two categories - one is febrile, and the other is afebrile seizures. Many underlying pathological reasons lead to abnormal neuronal activity without fever, e.g., genetic causes, traumatic or non-traumatic brain injury, infections, electrolyte imbalance, severe metabolic derangement, and neurodevelopmental and cardiovascular conditions [3]. Hence, it poses a clinical challenge in diagnostic evaluation and raises concerns about the etiology and likelihood of its recurrence.

To detect the underlying severe pathological condition in a pediatric patient who presented with a new-onset afebrile seizure may require an intervention, electroencephalogram (EEG) and neuroimaging is performed. EEG is recommended for a child presenting with a first afebrile seizure as a standard of care by the “American Academy of Neurology (AAN) and Child Neurology Society” [4]. The EEG is helpful to evaluate the risk of seizure recurrence, determine whether a seizure is focal or generalized, screen for focal abnormalities and the possible need for MRI, identify epilepsy syndrome classification, guide the choice of antiepileptics, and aid in prognosis. Whether an EEG should be done in the emergency room or as an in-patient or out-patient procedure is still debatable. Most studies have shown that the prevalence of abnormal neuroimaging in children presented with new-onset afebrile seizures is estimated to be 0–34% [5–7].

Studies regarding the risk of intracranial pathology in children with new-onset afebrile seizures are limited. Due to the lack of firm evidence, there continues to be controversy over performing emergent neuroimaging and EEG in children presenting with new-onset afebrile seizures and specific clinical findings that should trigger these investigations in the ED. This study aimed to determine the predictors of abnormal EEG and neuroimaging in children presenting to the emergency department with new-onset afebrile seizures.

## Methods

This single-center cross-sectional study was conducted at the Department of Emergency Medicine, Aga Khan University Hospital, Karachi, Pakistan, from July 01, 2019, to June 30, 2021. AKUH is a tertiary care hospital serving the city of Karachi, a city of over 20 million people. For this study, all patients aged one month to 18 years who presented to the ED with new-onset afebrile seizures were included. Children with congenital malformation syndrome, cerebral palsy, trauma, and those referred from other facilities were excluded. A total of 201 patients were included during the study period.

Data was collected on data collection forms via medical chart review and an online emergency medical record system. Junior residents were trained and responsible for data collection. Eligible patients were identified through electronically available triage presenting complaints. Demographic and clinical data, including age, gender, seizure type, duration of seizure, associated signs and symptoms, and disposition, were recorded from medical record files of the patients using a standardized form. Seizures were classified as generalized onset or focal onset according to the classification of the international league against epilepsy [8].

Data were analyzed by using SPSS version 21. Descriptive analysis was done, and frequencies with percentages were calculated for the above-mentioned variables. Multivariable regression analysis was applied to evaluate the significant association between the demographics, clinical examination findings, and abnormal EEG and neuroimaging findings. All tests were used assuming a 95% confidence level, considering a statistically significant value when p < 0.05.

## Results

Out of 201 participants, most patients were in the infantile age group (41.3%) and had almost an equal gender distribution. The most common type of seizure was generalized onset 152 (75.6%). Focal neurological deficits, drowsiness, and vomiting were the most frequently associated signs and symptoms. Most patients were admitted to inpatient care from the emergency department (73.6%) (Table 1).

**Table 1:** Baseline characteristics of children with abnormal EEG and Neuroimaging presenting to ED with new-onset afebrile seizures (n = 201)

Characteristics	Results *(n, %)*
Age Less than 1 year 1–5 years 5–10 years 10–18 years	83 (41.3) 39 (19.4) 51 (25.3) 28 (14)
Gender Female Male	101 (50.2) 100 (49.7)
Type of seizure Generalized onset Focal onset	152 (75.6) 49 (24.3)
Associated signs and symptoms Focal neurological deficit Drowsiness Vomiting Headache Diarrhea	22 (11) 14 (07) 09 (4.5) 04 (02) 03 (1.5)
Duration of the seizure Less than 5 min More than 5 min	188 (93.5) 13 (6.4)
Positive family history	01 (0.5)
Disposition from ER Admitted Discharged Left against medical advice	148 (73.6) 15 (7.5) 38 (19)

An EEG was performed on a total of 126 patients (62.7%), and out of these, 67 patients (53.1%) had abnormal findings. The most common abnormal EEG findings were sharp and slow waves (47.8%), followed by sharp waves (21%), and slow posterior dominant rhythm (15%) (Supplementary Table 1). Neuroimaging was performed in 102 patients (50.7%). Brain MRIs were performed on 70 patients (68.6%), brain CTs were performed on 24 patients (23.5%), and both a brain CT and MRI were done on 8 patients (7.8%). Abnormal neuroimaging findings were found in 37 patients (36.2%). EEG and neuroimaging were performed in 81 patients (40.3%) (Table 2). The most common abnormal neuroimaging findings were intracranial bleed (7.8%), followed by space-occupying lesion (2.9%), acute ischemic infarct (2.9%), and focal cortical dysplasia (2.9%). Only 19 (23.4%) patients with abnormal EEG had abnormal neuroimaging findings. (Supplementary Table 2).

**Table 2:** EEG findings in children presenting to the ED with new-onset afebrile seizures (n = 201)

Characteristics	Results *(n, %)*
EEG performed Yes No	126 (62.7) 75 (37.3)
Abnormal EEG findings	67 (53.1)
Neuroimaging performed CT Brain MRI Brain	70 (34.8) 24 (12.0)
Abnormal neuroimaging findings	37 (36.2%)

In the univariate analysis, male gender, age group of 5 to 10 years, seizure duration of more than 5 min, and focal neurological deficits were significantly associated with higher odds of abnormal EEG findings. In contrast, in the age group of 1 to 5 years, focal onset seizure, more than 5 min seizure duration, and focal neurological deficit were significantly associated with higher odds of abnormal neuroimaging findings. The patients with abnormal EEG who also had abnormal neuroimaging findings were 1.5 times (95% CI 0.57–3.93) higher odds. In multivariable analysis, the age group of 5 to 10 years and seizure duration of more than 5 min were significantly associated with higher odds of abnormal EEG findings. In contrast, only the focal onset of seizure was significantly associated with higher odds of abnormal neuroimaging findings (Table 3).

**Table 3:** Univariate and multivariable analysis for the predictors of abnormal EEG and Neuroimaging findings (n = 201)

Factors	Abnormal EEG				Abnormal Neuroimaging			
	**Univariate**		**Multivariable**		**Univariate**		**Multivariable**	
	**OR (95%CI)**	**P-value**	**OR (95%CI)**	**P-value**	**OR (95%CI)**	**P-value**	**OR (95%CI)**	**P-value**
Male Gender	2.74 [1.27–5.91]	0.01*	2.33 [0.82–6.58]	0.111	0.87 [0.4–1.89]	0.719		
Age Groups								
*1–5 years*	1.65 [0.62–4.39]	0.319	0.76 [0.2–2.79]	0.674	3.02 [1.24–9.16]	0.049*	2.13 [0.55–8.22]	0.272
*5–10 years*	3.97 [1.48–10.64]	0.006*	5.68 [1.64–19.75]	0.006*	1.86 [0.72–4.8]	0.202	1.54 [0.46–5.14]	0.487
*10–18 years*	2.78 [0.68–11.44]	0.157	4.09 [0.76–22.08]	0.102	1.08 [0.26–4.44]	0.911	1.12 [0.18–6.75]	0.905
Focal Onset	1.52 [0.63–3.46]	0.352			6.38 [2.3 -17.69]	< 0.001*	3.3 [1.44–11.01]	0.046*
Seizure Duration > 5 min	5.91 [1.3 -26.79]	0.021*	13.54 [2.08–88.14]	0.006*	2.72 [1.18–6.31]	0.02*	1.59 [0.56–4.55]	0.386
Associated signs and symptoms								
Focal Deficit	4.71 [1.32–16.8]	0.017*	3.2 [0.75–13.72]	0.117	2.9 [1.07–7.89]	0.037*	3.2 [0.75–13.72]	0.117
Drowsiness	0.51 [0.14–1.86]	0.304			0.76 [0.13–4.37]	0.762		
Vomiting	2.79 [0.32–24.68]	0.356			0.29 [0.03–2.6]	0.270		

## Discussion

This study evaluated the relationship between the patient characteristics, seizure pattern, and physical examination findings with abnormal neuroimaging and EEG results in pediatric patients who presented to the ED with their first afebrile seizure. Abnormal EEG and neuroimaging findings were observed in 53.1% and 36.2% of the total patients in these investigations. In this study, it was observed that age group of 5–10 years, seizure duration of ≥ 5 min and focal neurological deficits were significantly associated with abnormal EEG results, whereas in the age group of 1–5 years, a focal pattern of seizure, seizure duration of ≥ 5 min, and focal neurological deficits were significantly associated with abnormal neuroimaging findings.

EEG is an essential diagnostic test in evaluating a patient with new-onset afebrile seizures. It not only provides support for the diagnosis of seizure but also assists in determining whether to perform neuroimaging or not and impacts management decisions. Studies have shown that 18% and 56% of children presented with new-onset afebrile seizures have abnormal EEG results [9, 10]. This study also indicates EEG abnormalities in 53% of the patients.

Determining the factors associated with abnormal EEG are also crucial as performing EEG in all children presenting with new-onset afebrile seizure to the ED is cost-intensive. This study found various predictors of abnormal EEGs, including male gender, age group of 5–10 years, prolonged seizure, and focal neurological deficit. The results are consistent with other studies that also show that the age of more than five years and prolonged seizure is associated with abnormal EEG findings [11–15].

Neuroimaging is one of the essential investigations performed in patients with new-onset seizures. Its primary goal is to identify the underlying tissue abnormality or structural lesion that can explain the cause of the seizure. In this study, 36.2% of the patients had abnormal neuroimaging findings. The prevalence of abnormal neuroimaging findings varies among studies from 7 to 67% [16–20]. A study done by Minardi C. et al. suggests performing non-contrast CT head in all patients with the first clinical presentation of seizure and in clinical conditions that could increase the risk of complications [15].

It is crucial to identify the patients who need urgent neuroimaging based on the clinical history and physical examination findings to avoid the risk of radiation exposure, procedural sedation, and cost reasons. This study identified various predictors of abnormal neuroimaging, like age of 1–5 years, prolonged seizure duration, the focal onset of seizures, and focal neurological deficit on clinical examination. The results are consistent with previous studies that also identified similar predictors of abnormal neuroimaging findings [13, 14].

In this study, we didn’t find any significant abnormal neuroimaging findings in patients with abnormal EEG. This study’s results contrast with the studies in Cairo and India, which demonstrate that neuroimaging abnormalities were found more commonly in patients with abnormal EEG findings [13, 20].

### Limitations

This single-center cross-sectional study conducted in the emergency department may limit its generalizability. Around 37% and 46% of the patients did not have EEG and neuroimaging findings, so they could not be included in the analysis.

## Conclusion

The study identifies various predictors of abnormal EEG and neuroimaging findings in children presented to the emergency department with new-onset afebrile seizures. The age group of 5 to 10 years and seizure duration of more than 5 min was significantly associated with abnormal EEG findings. In contrast, the focal onset of seizure was mainly related to abnormal neuroimaging findings. The study emphasizes the need for a protocol regarding the performance of EEG and neuroimaging in children presenting to the ED with new-onset afebrile seizures that aid emergency physicians in the direction of appropriate management, thus ensuring a better quality of patient care and outcomes.

## Electronic supplementary material

Below is the link to the electronic supplementary material.

Supplementary Material 1

## Data Availability

Data and materials are available to the corresponding author, which can be shared at a reasonable request.

## Abbreviations

AAN: American Academy of Neurology
CT: Computed Tomography
ED: Emergency Department
EEG: Electroencephalogram
MRI: Magnetic Resonance Imaging
