# Supplementary material for: Predictors of abnormal electroencephalogram and neuroimaging in children presenting to the emergency department with new-onset afebrile seizures
Source: BMC Pediatr. 2022 Oct 27;22:619. doi: 10.1186/s12887-022-03668-6 (PMC9609244; doi:10.1186/s12887-022-03668-6)
Supplement: Supplementary file 1 — Supplementary Material 1 [file 12887_2022_3668_MOESM1_ESM.docx]

Supplementary table 1: Characteristics of EEG in pediatric patients

| **Characteristics** | **n (%)** |
| --- | --- |
| Sharp and slow waves | 32 (48) |
| Sharp waves | 14 (21) |
| Slow posterior dominant rhythm | 10 (15) |
| Slow waves | 04 (06) |
| Theta delta slowing | 04 (06) |
| Generalized delta burst | 03 (4.4) |

Supplementary table 2. Neuroimaging findings in pediatric patients

| **Characteristics** | **n (%)** |
| --- | --- |
| Intracranial bleed | 08 |
| Space occupying lesion | 03 |
| Acute infarct | 03 |
| Mesial temporal sclerosis | 03 |
| Diffuse cerebral edema | 03 |
| Encephalomalacia | 03 |
| Periventricular leukomalacia | 03 |
| Focal cortical dysplasia | 03 |
| Empyema | 01 |
| Lissencephaly | 01 |
| Delayed myelination | 01 |
| Leukodystrophy | 01 |
| Hypogenetic corpus callosum | 01 |
| Hydrocephalus | 01 |
